# Long-term use of renin-angiotensin-system inhibitors after acute myocardial infarction is not associated with survival benefits: Analysis of data from the Korean acute myocardial infarction registry-national institutes of health registry

**DOI:** 10.3389/fcvm.2022.994419

**Published:** 2022-08-31

**Authors:** Chan Soon Park, Han-Mo Yang, Jeehoon Kang, Jung-Kyu Han, Kyung Woo Park, Hyun-Jae Kang, Bon-Kwon Koo, Ki-Bae Seung, Kwang Soo Cha, In-Whan Seong, Seung-Woon Rha, Myung Ho Jeong, Hyo-Soo Kim

**Affiliations:** ^1^Department of Internal Medicine, Seoul National University Hospital, Seoul, South Korea; ^2^Cardiology Division, Department of Internal Medicine, College of Medicine, The Catholic University of Korea, Seoul, South Korea; ^3^Department of Internal Medicine, Pusan National University Hospital, Busan, South Korea; ^4^Department of Internal Medicine, College of Medicine, Chungnam National University Hospital, Chungnam National University, Daejeon, South Korea; ^5^Department of Internal Medicine, College of Medicine, Kyungpook National University, Daegu, South Korea; ^6^Department of Internal Medicine and Heart Center, Chonnam National University Hospital, Gwangju, South Korea

**Keywords:** acute myocardial infarction, renin-angiotensin-system inhibitor, angiotensin converting enzyme inhibitor, angiotensin II receptor blocker, mortality, prognosis

## Abstract

**Introduction:**

Renin-angiotensin-system inhibitors (RASi) have shown survival benefits after acute myocardial infarction (MI), but the role of routine long-term use of RASi remains unclear. Thereby, we explored the therapeutic effects of RASi medication at 1-year follow-up from acute MI.

**Methods:**

Using the nationwide Korea Acute Myocardial Infarction Registry-National Institutes of Health (KAMIR-NIH) registry, we included and analyzed 10,822 subjects. Patients were stratified into those taking RASi at 1-year follow-up (*n* = 7,696) and those not taking RASi at 1-year follow-up (*n* = 3,126). Patients were followed up for 2-years from the 1-year follow-up; 2-year all-cause mortality and cardiac mortality were analyzed as primary and secondary outcomes, respectively.

**Results:**

The use of RASi at 1-year follow-up was not associated with decreased all-cause mortality (log-rank *P* = 0.195) or cardiac mortality (log-rank *P* = 0.337). In multivariate analyses, RASi medication at 1-year follow-up did not reduce all-cause mortality (*P* = 0.758) or cardiac mortality (*P* = 0.923), while RASi medication at discharge substantially reduced 1-year all-cause and cardiac mortality. Treatment with either an angiotensin-converting enzyme inhibitor or angiotensin II receptor blocker at 1-year follow-up did not show survival benefits from 1-year follow-up, respectively. The use of RASi at 1-year follow-up did not show a prognostic interaction between previous history of chronic kidney disease, post-MI acute heart failure, concomitant use of beta-blockers at 1-year follow-up, or 1-year LVEF.

**Conclusion:**

Acute MI patients taking RASi at 1-year follow-up were not associated with improved 2-year all-cause mortality or cardiac mortality from the 1-year follow-up. This study provides valuable information regarding tailored medication strategy after acute MI.

**Clinical trial registration:**

[www.ClinicalTrials.gov], identifier [KCT0000863].

## Introduction

Owing to its global disease burden (1, 2), various attempts have been made to manage acute myocardial infarction (MI). Although recent studies have reported that both age-standardized incidence and mortality of acute MI have been gradually decreasing (2–4), acute MI still accounts for an unacceptably high mortality rate (1, 3). Therefore, there is an unmet demand to make the clinical outcomes of patients with acute MI better.

Activation of the renin-angiotensin-system (RAS) could have detrimental effects on the cardiovascular system by causing oxidative stress, endothelial dysfunction, and inflammation (5). Based on this theoretical background, the use of angiotensin-converting enzyme inhibitors (ACEi) has successfully proven its therapeutic benefits, such as reducing mortality and recurrent MI in acute MI patients (6, 7). Angiotensin receptor II blocker (ARB) medication also showed its equivalent therapeutic implication, as compared to that of ACEi (8). Accordingly, the use of renin-angiotensin-system inhibitors (RASi), including both ACEi and ARB, is recommended for acute MI patients, especially in those with reduced left ventricular ejection fraction (LVEF) (9–12). However, the benefits of routine long-term use of RASi remain elusive (13, 14); it is unclear how long ACEi should be prescribed after acute MI in current guidelines. Similarly, long-term use of beta-blockers after acute MI remains unclear; we found that beta-blockers at 1-year follow-up are associated with better prognosis in patients with 1-year LVEF < 50%, but not in those with 1-year LVEF ≥ 50% (15).

We postulated that the therapeutic effects of RASi might be attenuated in acute MI patients who underwent revascularization and received medical treatment for a long time. We also wondered whether the beneficial effects of long-term use of RASi might be affected by 1-year left LVEF or concomitant beta-blocker therapy. To answer these questions, we aimed to investigate the clinical outcomes of long-term RASi use in a large, prospective, multicenter study of patients with acute MI.

## Materials and methods

### Study population and data collection

**FIGURE 1:**
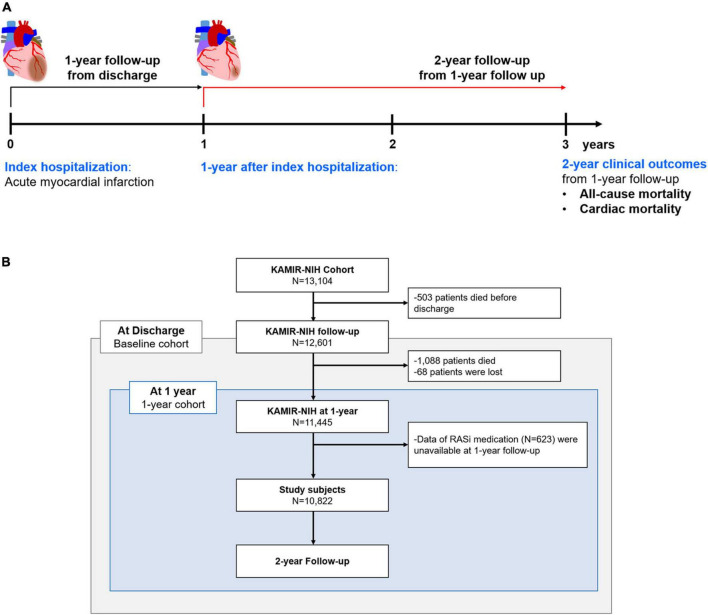
Study population. Study flow chart **(A)** and patient demographics based on the flow chart **(B)** are illustrated. RASi, renin-angiotensin-system inhibitor; KAMIR-NIH, Korean Acute Myocardial Infarction-National Institutes of Health.

We analyzed the Korean Acute Myocardial Infarction Registry-National Institutes of Health (KAMIR-NIH) registry, a prospective, multicenter registry. The detailed design and preliminary results were published elsewhere (16). In brief, 13,104 consecutive patients hospitalized for acute MI in 20 tertiary university hospitals in the Republic of Korea were enrolled between November 2011 and December 2015. There were no exclusion criteria for enrolment in the KAMIR-NIH registry; all participating hospitals were eligible for primary percutaneous coronary intervention. Among these, we mainly analyzed participants who were alive at 1-year and whose data regarding their 1-year medication were available. All patients were treated according to the clinical guidelines of each participating hospital, and the use of RASi was considered unless contraindicated (9–12). To compare the benefits of RASi at discharge and those of RASi at 1-year, we additionally analyzed the effects of RASi at discharge in the baseline cohort, which is composed of patients alive at discharge from the index hospitalization (Figure 1A). Among 11,513 patients alive at 1-year, data on the RASi medication was available in 10,822 subjects (94.0%). The study protocol of the KAMIR-NIH registry was approved by the Institutional Review Board of each participating hospital and was conducted according to the principles of the Declaration of Helsinki.

### Study variables and definitions

Based on their medication history, patients were classified into those without RASi medication and those with RASi medication. In terms of medication, data regarding the use of RASi, beta-blockers, and statins were collected. Prescription of either an ACEi or an ARB was defined as use of RASi. Medication history at discharge and during follow-up (1, 2, and 3-year follow-up) was recorded in the KAMIR-NIH registry. Regarding LVEF, patients were classified into those with 1-year LVEF < 50% and those with 1-year LVEF ≥ 50%.

The primary and secondary outcomes were 2-year all-cause mortality and cardiac mortality from 1-year follow-up, according to the use of RASi at 1-year follow-up, respectively (Figure 1B). All deaths were considered cardiac unless an undisputed non-cardiac cause was present. To compare the benefits of RASi at discharge and those of RASi at 1-year follow-up, we also evaluated all-cause and cardiac mortality according to RASi at discharge among the baseline cohort. Mortality data were obtained and verified *via* the Ministry of the Interior and Safety, a government agency of the Republic of Korea.

### Statistics

Descriptive data were expressed as numbers and frequencies for categorical variables, and as means ± standard deviations for continuous variables. We performed the unpaired Student’s *t*-test for continuous variables and the χ^2^-test (or Fisher’s exact test when any expected cell count was <5 for a 2 × 2 table) for categorical variables, for comparison between groups. The chronological trend of the clinical outcomes was presented as Kaplan-Meier curves and compared according to the medication. The log-rank test was used to compare the differences in clinical prognosis. A multivariable Cox proportional hazard regression model was used to determine the independent predictors of all-cause mortality. Following variables were included for the multivariable model as they had significant predictive values in cardiovascular diseases: age, sex, body mass index, diabetes mellitus, chronic kidney disease, previous MI, previous heart failure, smoking status, ST segment elevation MI (STEMI), completeness of revascularization, beta-blocker medication at 1-year follow-up, and statin medication at 1-year follow-up.

Statistical significance was acknowledged when two-sided *P*-values of < 0.05. Statistical tests were performed using IBM SPSS version 23 (SPSS Inc., Chicago, IL, United States) and R programming (version 3.6.1; The R Foundation for Statistical Computing, Vienna, Austria).

## Results

### Demographic features of study subjects

Among all subjects enrolled in the KAMIR-NIH registry, 503 patients died before discharge; an additional 1,088 patients died before the 1-year follow-up period. After excluding those who were lost or whose data regarding medication were not available at 1-year follow-up, 10,822 subjects were defined and analyzed as a 1-year cohort in this study. Among included, 9,689 (91.2%) subjects were treated with percutaneous coronary intervention, and 186 (1.7%) patients were treated with coronary artery bypass graft surgery. According to their RASi prescription at 1-year follow-up, patients were classified as those taking RASi (71.1%, *n* = 7,696) and those not taking RASi (28.9%, *n* = 3,126).

The clinical features according to 1-year RASi medication are presented in Table 1. Briefly, patients taking RASi at 1-year were younger with a history of hypertension and were more frequently diagnosed with STEMI. They also presented a higher prescription rate of beta-blockers and statins than their counterparts. In contrast, patients not taking RASi at 1-year follow up showed more frequent history of chronic kidney disease. There was no significant difference in the history of diabetes mellitus, dyslipidemia, or MI between the two groups. Demographic data of the baseline cohort are presented in Supplementary Table 1.

**TABLE 1:** Clinical characteristics based on use of RAS-inhibitor at 1-year follow-up.

	Without RASi (*n* = 3,126)	With RASi (*n* = 7,696)	*P*-value
***At index admission***			
**Demographic data**			
Age (years)	63.5 ± 12.2	62.7 ± 12.3	0.001
Men (%)	2,344 (75.0)	5,783 (75.1)	0.862
BMI (kg/m^2^)	23.7 ± 3.3	24.3 ± 3.2	<0.001
**Past medical history (%)**			
Hypertension	1,298 (41.5)	4,092 (53.2)	<0.001
Diabetes mellitus	825 (26.4)	2,108 (27.4)	0.289
Dyslipidemia	353 (11.3)	912 (11.9)	0.413
Chronic kidney disease	444 (14.2)	936 (12.2)	0.004
Myocardial infarction	230 (7.4)	558 (7.3)	0.846
Congestive heart failure	36 (1.2)	98 (1.3)	0.612
Cerebrovascular accident	190 (6.1)	468 (6.1)	0.996
Current smoking	1,178 (38.7)	3,182 (42.4)	<0.001
**Characteristics of lesion and PCI (%)**			
STEMI	1,395 (44.6)	3,834 (49.8)	<0.001
Complete revascularization	1,898 (71.3)	5,009 (69.6)	0.104
**Peak cardiac enzyme levels**			
CK-MB (ng/mL)	45.2 (8.8–160.5)	48.7 (9.2–167.7)	0.188
Troponin I (ng/mL)	14.5 (2.1–40.0)	18.0 (3.0–50.0)	0.093
**Echocardiography**			
LVEF (%)	53.0 ± 10.6	52.5 ± 10.8	0.009
LVEF < 50% (%)	1,032 (34.2)	2,751 (36.6)	0.018
**Physical exam at discharge**			
SBP (mmHg)	129.3 ± 27.3	132.6 ± 29.1	<0.001
DBP (mmHg)	78.4 ± 16.4	80.2 ± 17.7	<0.001
HR (beats per min)	77.2 ± 18.4	78.1 ± 18.4	0.013
**Medication at discharge (%)**			
Beta-blocker	2,408 (77.0)	6,740 (87.6)	<0.001
RASi	1,715 (54.9)	6,893 (60.7)	<0.001
Statin	2,907 (93.0)	7,276 (94.5)	0.002
***At 1-year follow-up***			
**Physical exam**			
SBP (mmHg)	122.6 ± 16.1	125.8 ± 16.7	<0.001
DBP (mmHg)	72.4 ± 11.3	74.1 ± 12.2	<0.001
HR (beats per min)	69.9 ± 19.7	67.5 ± 21.0	<0.001
**Echocardiography**			
LVEF (%)	55.5 ± 10.5	55.5 ± 10.7	0.873
LVEF < 50% (%)	273 (24.8)	728 (25.0)	0.888
**Medication (%)**			
Beta-blocker	2,042 (65.3)	6,313 (82.0)	<0.001
Statin	2,724 (87.1)	7,258 (94.3)	<0.001

BMI, Body mass index; CK-MB, Creatine kinase-myocardial band; DBP, Diastolic blood pressure; HF, Heart failure; HR, Heart rate; LVEF, Left ventricular ejection fraction; RASi, Renin-angiotensin-system inhibitor; SBP, Systolic blood pressure; STEMI, ST-segment elevation myocardial infarction.

### Clinical outcomes or renin-angiotensin-system inhibitors medication at 1-year follow-up

**FIGURE 2:**
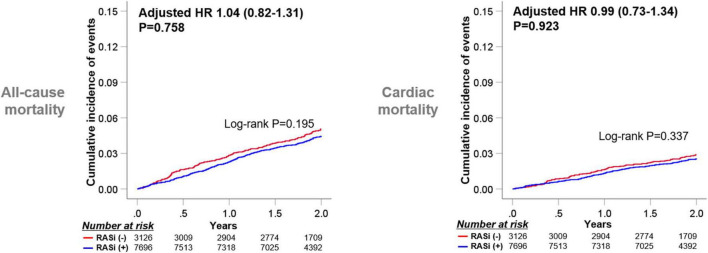
Clinical outcomes according to RASi medication at 1-year follow-up. Kaplan-Meier survival curves for 2-year clinical outcomes from 1-year follow-up according to RASi medications at 1-year follow-up are presented. HR, Hazard ratio; RASi, renin-angiotensin-system inhibitor.

During the 2-year follow-up from 1-year after index hospitalization, 475 patients (4.4%) died. Figure 2 presents Kaplan-Meier survival curves for all-cause mortality and cardiac mortality according to the use of RASi at 1-year follow-up. For both clinical outcomes, the use of RASi at 1-year follow-up was not associated with improved clinical outcomes (log-rank *P* = 0.195 and log-rank *P* = 0.337, respectively). After adjusting for significant covariates using Cox proportional regression analysis, RASi at 1-year follow-up still did not improve clinical outcomes for all-cause mortality (hazard ratio [HR] 1.04, 95% confidence interval [CI] 0.82–1.31, *P* = 0.758) and cardiac mortality (HR 0.99, 95% CI 0.73–1.34, *P* = 0.923). Diminished prognostic benefits of RASi at 1-year follow-up were observed regardless of the use of ACEi or ARB (Supplementary Figure 1). In contrast, when we analyzed the prognostic impact of RASi at discharge with the baseline cohort, the use of RASi proved substantial survival benefits in line with previous reports (Supplementary Figure 2) (6, 7). RASi at discharge was associated with decreased all-cause mortality in both univariate (log-rank *P* < 0.001) and multivariate analyses (HR 0.78, 95% CI 0.61–0.99, *P* = 0.039) from index hospitalization. RASi at discharge also reduced cardiac mortality in both univariate (log-rank *P* = 0.006) and multivariate analyses (HR 0.67, 95% CI 0.50–0.89, *P* = 0.006).

**FIGURE 3:**
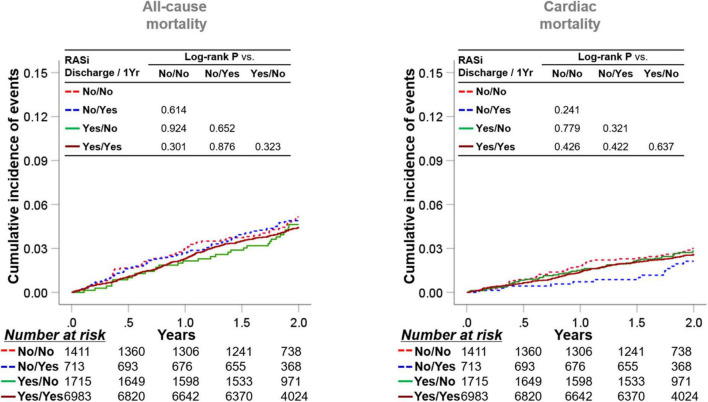
Implication of RASi stratified by medication at discharge and at 1-year follow-up. Patients were categorized into four subgroups according to the RASi medication at discharge and at the 1-year follow-up. Kaplan-Meier survival curves for 2-year all-cause mortality and cardiac mortality from the 1-year follow-up are illustrated. RASi: renin-angiotensin-system inhibitor.

As the medication history of patients who took RASi or not could be altered during the 1-year follow-up after discharge, we stratified the subjects into four groups according to RASi medication at discharge and 1-year follow-up (Figure 3). Taking RASi at 1-year follow-up failed to reduce all-cause mortality in patients not taking RASi at discharge (log-rank *P* = 0.614), as well as those taking RASi at discharge (log-rank *P* = 0.323). Similarly, the use of RASi at 1-year follow-up was not related to decreased cardiac mortality, regardless of RASi medication at discharge.

**FIGURE 4:**
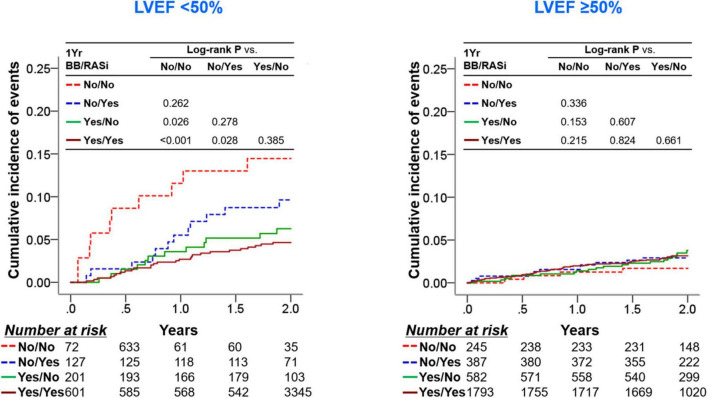
Clinical outcomes according to use of RASi and BB at 1-year follow-up and 1-year LVEF. In each group of patients with a 1-year LVEF < 50% and with a 1-year LVEF ≥ 50%, patients were stratified into four subgroups according to the RASi medication and beta-blocker medication at the 1-year follow-up. Kaplan-Meier survival curves for 2-year all-cause mortality from the 1-year follow-up are shown. BB, beta-blocker; LVEF, left ventricular ejection fraction; RASi, renin-angiotensin-system inhibitor.

We showed the differential therapeutic effects of beta-blockers at 1-year follow-up according to 1-year LVEF (15). In contrast, the use of RASi demonstrated reduced cardiac mortality but similar all-cause mortality in patients with 1-year LVEF <50% in the univariate analysis. After adjusting covariates, both groups of patients with 1-year LVEF <50% and with 1-year LVEF ≥50% did not show all-cause or cardiac mortality risk reduction (Supplementary Figure 3). We then stratified the subjects according to the use of RASi and beta-blockers at 1-year follow-up and 1-year LVEF (Figure 4). In patients with 1-year LVEF <50%, the use of beta-blockers at 1-year follow-up showed survival benefits in those taking or not taking RASi at 1-year follow-up (log-rank *P* = 0.028 and log-rank *P* = 0.026, respectively), while RASi medication at 1-year follow-up failed to show survival benefits in those taking or not taking beta-blockers at 1-year follow-up (log-rank *P* = 0.385 and log-rank *P* = 0.262). Regarding patients with 1-year LVEF ≥50%, neither the use of RASi nor beta-blockers at 1-year follow-up were associated with improved clinical outcomes.

In contrast, the use of RASi and beta-blockers at discharge significantly decreased 1-year all-cause mortality and cardiac mortality separately in the baseline cohort, with similar therapeutic benefits of RASi and beta-blocker medication at discharge. Concomitant use of RASi and beta-blockers at discharge further improved clinical outcomes, as compared to the single use of medication (Supplementary Figure 4). Interestingly, synergistic survival benefits of RASi and beta-blockers at discharge were not significantly observed during the first 3 months (Supplementary Figure 5).

### Subgroup analyses

**FIGURE 5:**
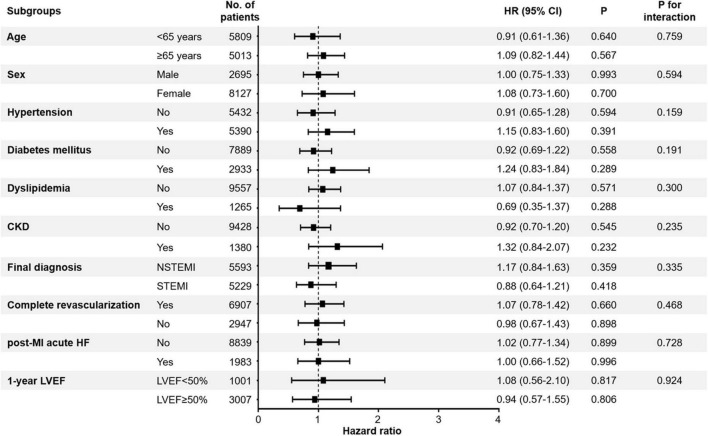
Association between all-cause mortality and use of RASi. The effects of RASi at 1-year follow-up for 2-year all-cause mortality in the exploratory subgroups were analyzed. CI, confidence interval; HF, heart failure; HR, hazard ratio; LVEF, left ventricular ejection fraction; MI, myocardial infarction; RASi, renin-angiotensin-system inhibitor; STEMI, ST-segment elevation myocardial infarction; NSTEMI, non-ST-segment elevation myocardial infarction.

We performed exploratory analyses, including subgroups based on age, sex, previous history of hypertension, diabetes mellitus, dyslipidemia, chronic kidney disease, STEMI, complete revascularization, post-MI acute heart failure, and 1-year LVEF (Figure 5). RASi at 1-year generally showed a lack of therapeutic implications, with no significant association.

## Discussion

In this study, we explored the therapeutic implications of long-term RASi use after acute MI. We stratified patients according to the use of RASi at 1-year follow-up and investigated survival benefits using the KAMIR-NIH, a well-controlled nationwide, prospective registry for acute MI. The major findings of this study are as follows: (1) Use of RASi at 1-year follow-up was not associated with survival benefits as our first hypothesis, while use of RASi at index hospitalization showed substantial survival benefits; (2) neither use of ACEi nor ARB at 1-year follow-up showed survival benefits; and (3) lack of prognostic benefits of RASi at 1-year follow-up was consistently observed, regardless of RASi medication history at index hospitalization, concomitant use of beta-blockers at 1-year follow-up, or 1-year LVEF, which was contradictory to our second hypothesis.

The implications of RAS have been intensively studied in the cardiovascular field over the past decades. It plays a significant role in regulating blood pressure and electrolyte balance (17). However, it also plays pivotal roles in various pathologies, such as aggravating hypertension, heart failure, and MI by provoking endothelial dysfunction, tissue remodeling, oxidative stress, and inflammation (18). Although renin itself has prognostic value in predicting cardiovascular effects, including MI (19, 20), most of the clinical importance of RAS in the cardiovascular fields is attributable to the cardioprotective effects of RASi, which comprises ACEi and ARB.

Regarding acute MI, both ACEi and ARB successfully proved their survival benefits. In various randomized trials, the use of ACEi significantly reduced fatal and non-fatal major cardiovascular events after acute MI (6, 7, 21, 22); ARB use also showed substantial therapeutic benefits equivalent to that of ACEi (8, 23). Not only were the benefits of RASi medication remarkable in acute MI patients with heart failure and left ventricular systolic dysfunction (24, 25), but previous studies also showed that the use of RASi could be beneficial in all MI patients, unless contraindicated (25, 26). Accordingly, the use of RASi became one of the key therapeutic approaches in managing MI patients. However, with early revascularization with percutaneous coronary intervention, the use of high-dose statins, and the advancement of antiplatelet regimens, the clinical importance of RASi has decreased. Nonetheless, using RASi is still advocated in the current guidelines unless contraindicated and could have its benefits in managing acute MI patients.

In contrast to robust evidence of prompt RASi medication after acute MI, the role of routine long-term RASi use remains unclear (Table 2). Braunwald et al. showed that the use of RASi was not associated with therapeutic benefits in patients with stable coronary heart disease and standard medical therapy in the Prevention of Events with Angiotensin Converting Enzyme Inhibition (PEACE) Trial (13). In addition, a previous report has also presented that ARB medication at discharge did not improve prognosis from 2 to 5 years after discharge, although patients taking ARB at discharge seemed to have better 5-year mortality compared to those not taking RASi (14). However, this study did not collect medication data at 2-year follow-up after acute MI. As the medication status of RASi could be altered during the follow-up, we stratified acute MI patients according to RASi medication at 1-year follow-up and found that the use of RASi at 1-year follow-up was not related to improved survival from 1-year follow-up.

**TABLE 2:** Comparison of studies about the limited role of RASi medication.

	Study design and study population	Number of subjects	Country	Index time of RASi medication	Follow-up duration	Outcomes
Park et al. (ours)	-Prospective registry -Patients with acute MI	10,822	Republic of Korea	1-year follow up after acute MI	2 from 1-year follow-up	RASi at 1-year follow-up was not associated with reduced all-cause mortality (*P* = 0.758) or reduced cardiac mortality (*P* = 0.923).
Braunwald et al. (13)	-Randomized controlled trial -Patients with stable coronary artery disease	8,290	United States, Canada, and Italy	At randomization	Median 4.8 years	No prognostic difference in death from cardiovascular causes, myocardial infarction, or coronary revascularization was observed between the trandolapril group and the placebo group (*P* = 0.43).
Hara et al. (14)	-Prospective registry -Patients with acute MI	9,025	Japan	At discharge	3 from 2-year follow-up (landmark analysis)	The survival estimate of the ARB group was comparable to that of the no RASi group (*P* = 0.72), while that of ACEi group was better (*P* = 0.004)

MI, myocardial infarction, RASi, Renin-angiotensin-system inhibitor.

Our study provides valuable information on treatment strategies for managing patients with acute MI. In contrast to the substantial survival benefits of initiating RASi medication within a short time after acute MI, we found that the administration of RASi at 1-year follow-up was not related to improved survival benefits from 1-year after acute MI. Additionally, the use of RASi at 1-year follow-up did not show prognostic interaction with use of beta-blockers at 1-year follow-up or 1-year LVEF. This does not mean that RASi should be discontinued after 1-year after acute MI, but suggests that RASi medications could be reconsidered carefully in patients who have clinical risks, that is, patients with marginal blood pressure, at 1-year follow-up.

Recently, there was a report that the use of sacubitril-valsartan was not associated with a lower incidence of death from cardiovascular causes or incident heart failure than the use of ramipril in acute MI patients complicated by a reduced LVEF (27). It might be cautiously speculated that early revascularization with percutaneous coronary intervention, intense antiplatelet agent regimen, and high-dose statins leave little room for further prognostic improvement with intensified neurohumoral inhibition (28). In this regard, it could not be counterintuitive that long-term use of RASi at 1-year follow-up was not associated with improved 2-year all-cause mortality or cardiac mortality from the 1-year follow-up.

This study has several limitations. First, although the KAMI-NIH registry is a well-designed, nationwide, prospective registry, it is an analysis of a prospective cohort study, rather than a randomized trial. Although all patients were treated using the clinical guidelines of each participating tertiary university hospital (9–12), there could be unmeasured confounders that may influence the results. Second, substantial number of patients did not have echocardiographic data at 1-year follow-up. Though use of RASi at 1-year follow-up failed to provide survival benefits in both overall 1-year cohort (Figure 2) and in patients who had 1-year echocardiographic data (Supplementary Figure 3), there might be a selection bias for performing 1-year echocardiography. Third, as our study included only East Asians, extrapolating these results to other countries and ethnicities might require careful consideration. Fourth, we could not collect the dosage of RASi medications at 1-year follow-up; therefore, we could not explore the dosage effect of the long-term use of RASi.

## Conclusion

Long-term use of RASi after 1-year follow-up was not associated with improved all-cause mortality and cardiac mortality, while RASi medications at index hospitalization proved substantial survival benefits. Therefore, this study might provide valuable information regarding the proper duration of RASi after acute MI.

## Data availability statement

For reasonable request, data would be available through approval of the corresponding author.

## Ethics statement

The studies involving human participants were reviewed and approved by the Institutional Review Board of each participating hospital. The patients/participants provided their written informed consent to participate in this study.

## Author contributions

CSP and H-MY conceptualized the study and were responsible for review and editing. CSP drafted the manuscript. CSP, H-MY, JK, J-KH, KWP, H-JK, B-KK, K-BS, KSC, I-WS, S-WR, MHJ, and H-SK contributed to the data acquisition. CSP, H-MY, JK, J-KH, KWP, H-JK, B-KK, and H-SK interpreted the data. K-BS, KSC, I-WS, S-WR, and MHJ supervised the project. All authors approved the submitted manuscript.
