# Supplementary material for: Long-term use of renin-angiotensin-system inhibitors after acute myocardial infarction is not associated with survival benefits: Analysis of data from the Korean acute myocardial infarction registry-national institutes of health registry
Source: Front Cardiovasc Med. 2022 Aug 31;9:994419. doi: 10.3389/fcvm.2022.994419 (PMC9471088; doi:10.3389/fcvm.2022.994419)
Supplement: Supplementary file 1 [file Data_Sheet_1.docx]

**Supplemental Material**


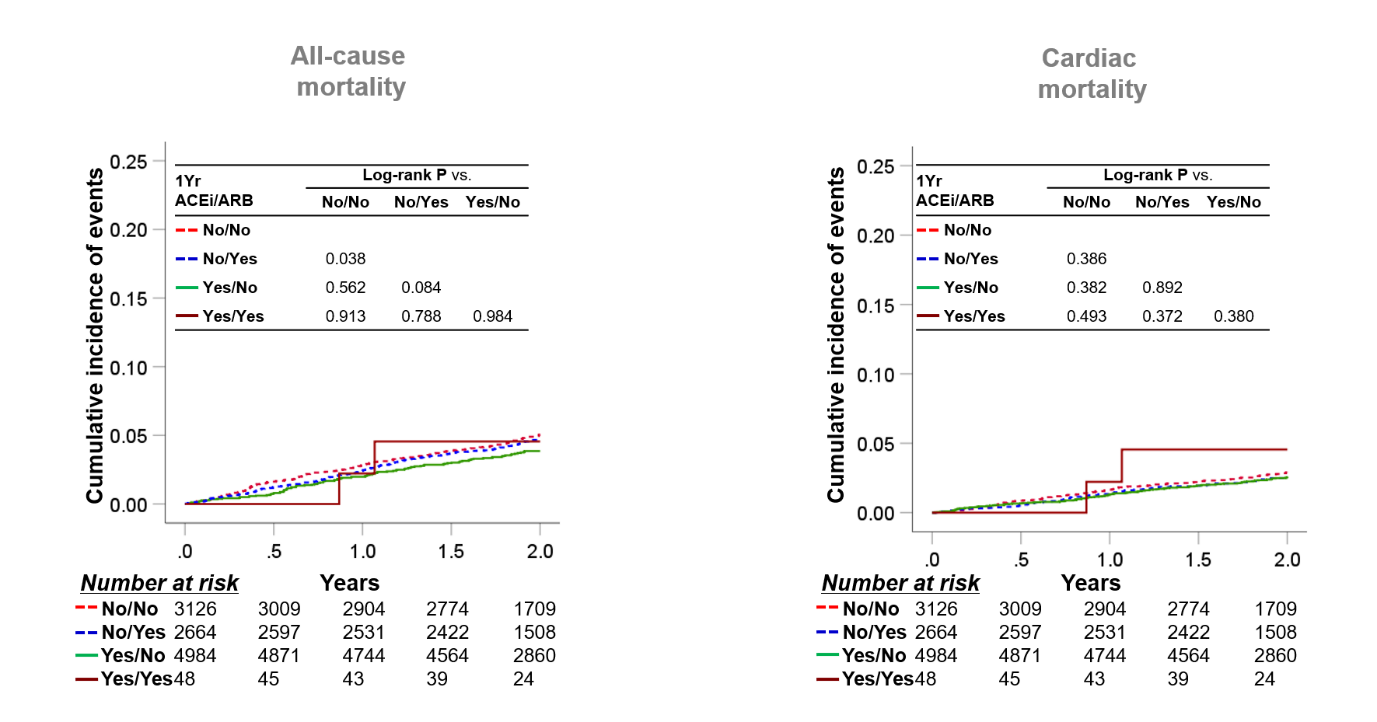


**Supplemental Figure 1. Clinical outcomes according to use of ACEi and ARB at 1-year follow-up**

Kaplan-Meier survival curves for 2-year all-cause mortality from 1-year follow-up are presented.

ACEi: angiotensin converting enzyme inhibitor, ARB, angiotensin II receptor blocker


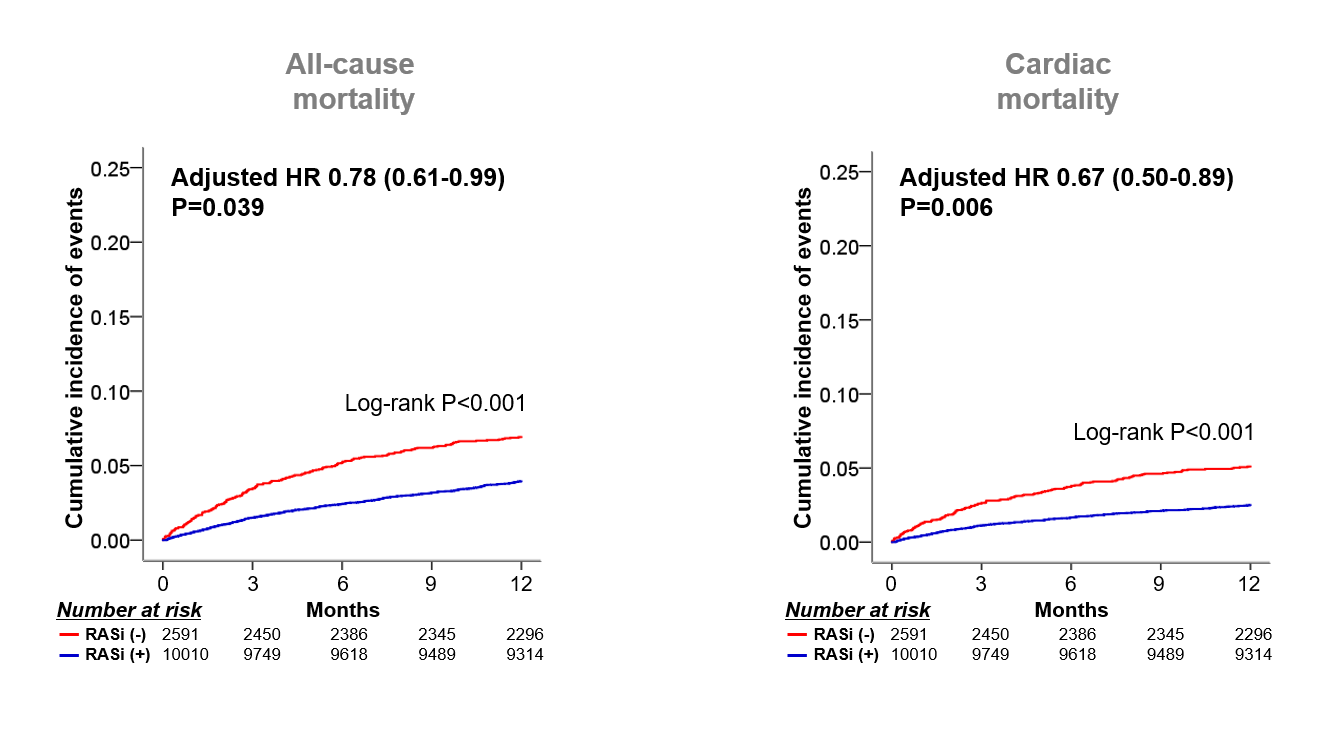


**Supplemental Figure 2. 1-year clinical outcomes according to RASi medication at discharge from index hospitalization**

Kaplan-Meier survival curves for 1-year post-discharge all-cause mortality and cardiac mortality are presented.

HR: hazard ratio, LVEF: Left ventricular ejection fraction, RASi: renin-angiotensin-system inhibitor


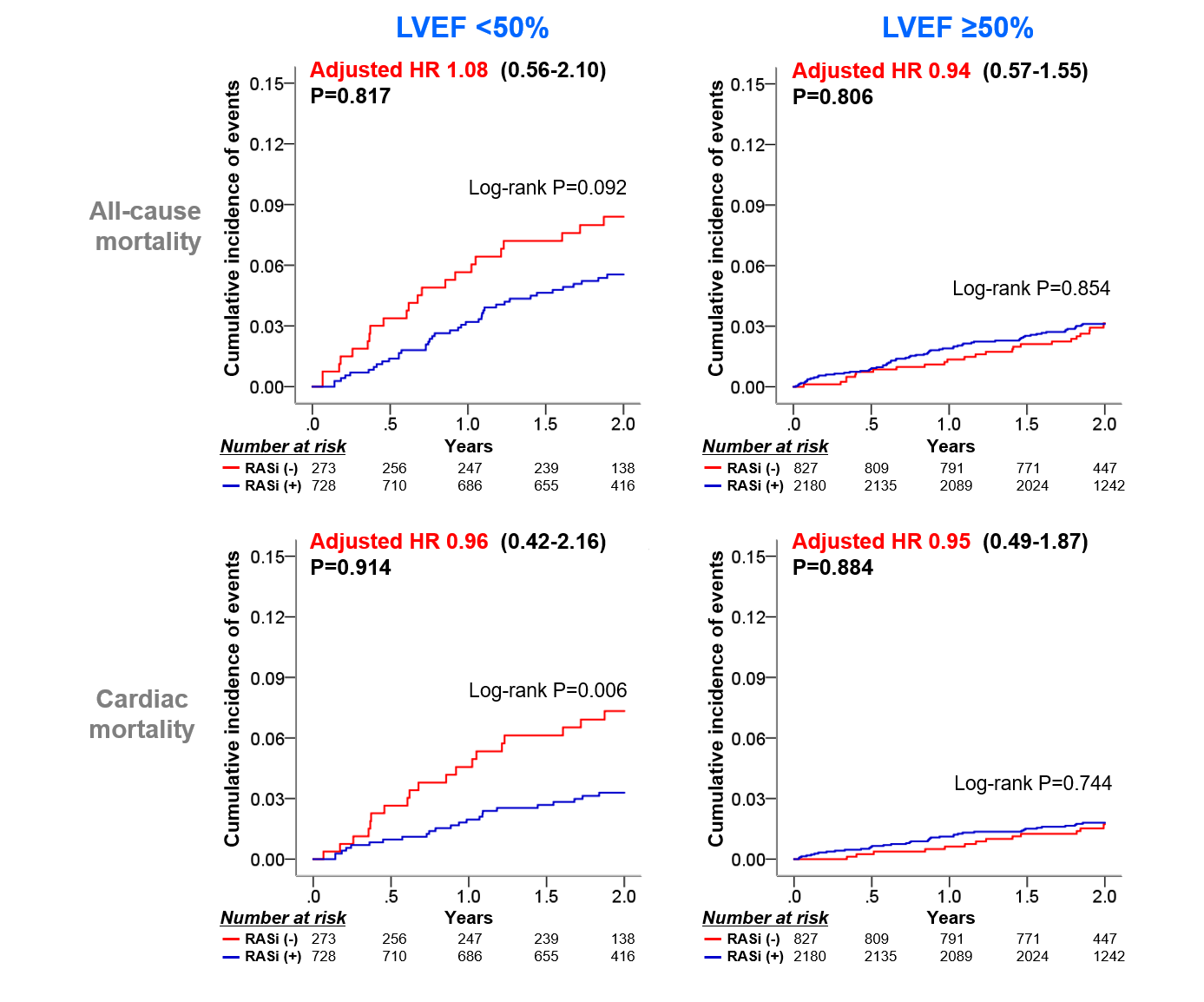


**Supplemental Figure 3. Clinical outcomes according to the RASi medication at 1-year follow-up and 1-year LVEF**

Kaplan-Meier survival curves for 2-year clinical outcomes according to RASi medication at 1-year and 1-year LVEF are presented. All-cause mortality and cardiac mortality were analyzed for 2-year clinical outcomes from 1-year follow-up.

HR: hazard ratio, LVEF, left ventricular ejection fraction, RASi: renin-angiotensin-system inhibitor


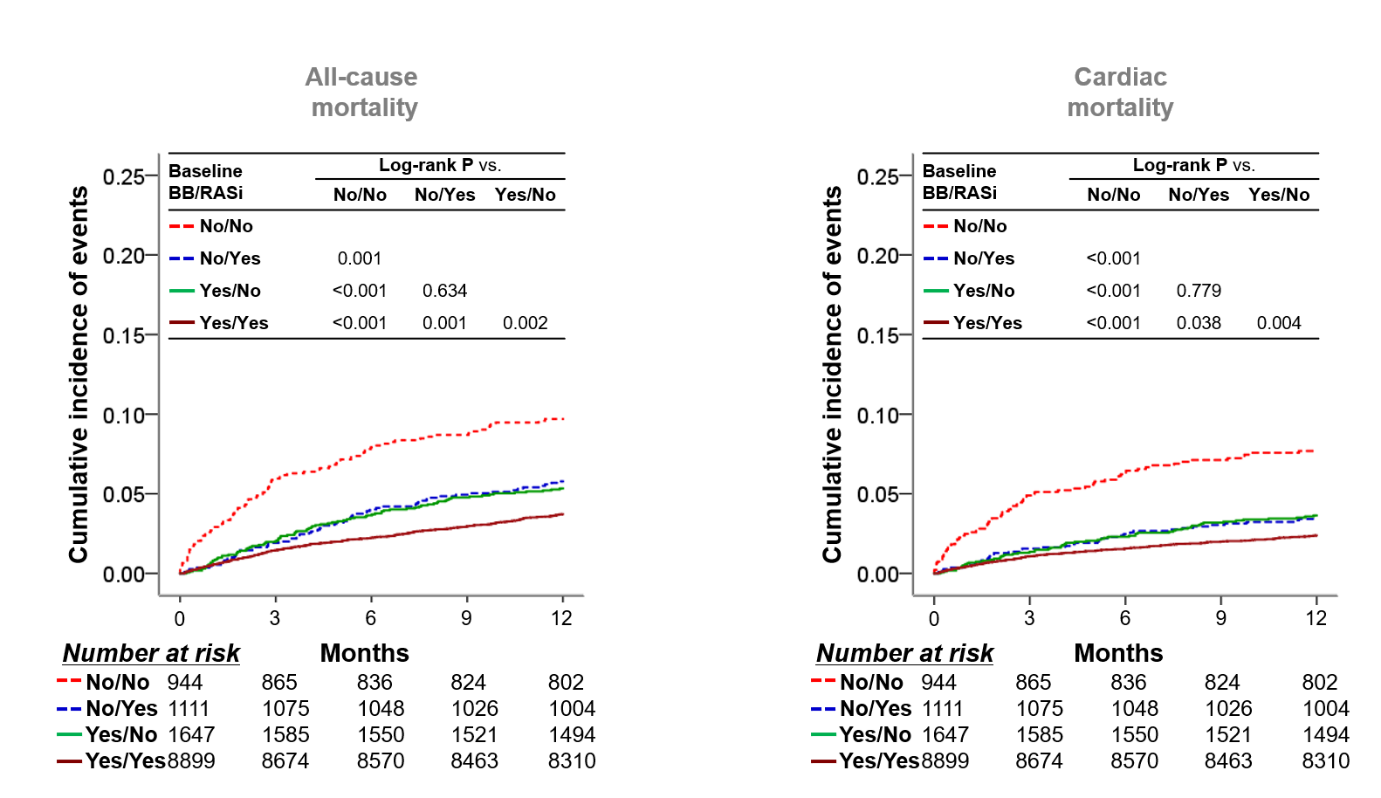


**Supplemental Figure 4. 1-year clinical outcomes according to RASi medication and beta-blocker medication at discharge from index hospitalization**

Kaplan-Meier survival curves for 1-year post-discharge all-cause mortality and cardiac mortality are presented. All-cause mortality and cardiac mortality were analyzed for 2-year clinical outcomes from 1-year follow-up.

BB: beta-blocker, HR: hazard ratio, RASi: renin-angiotensin-system inhibitor


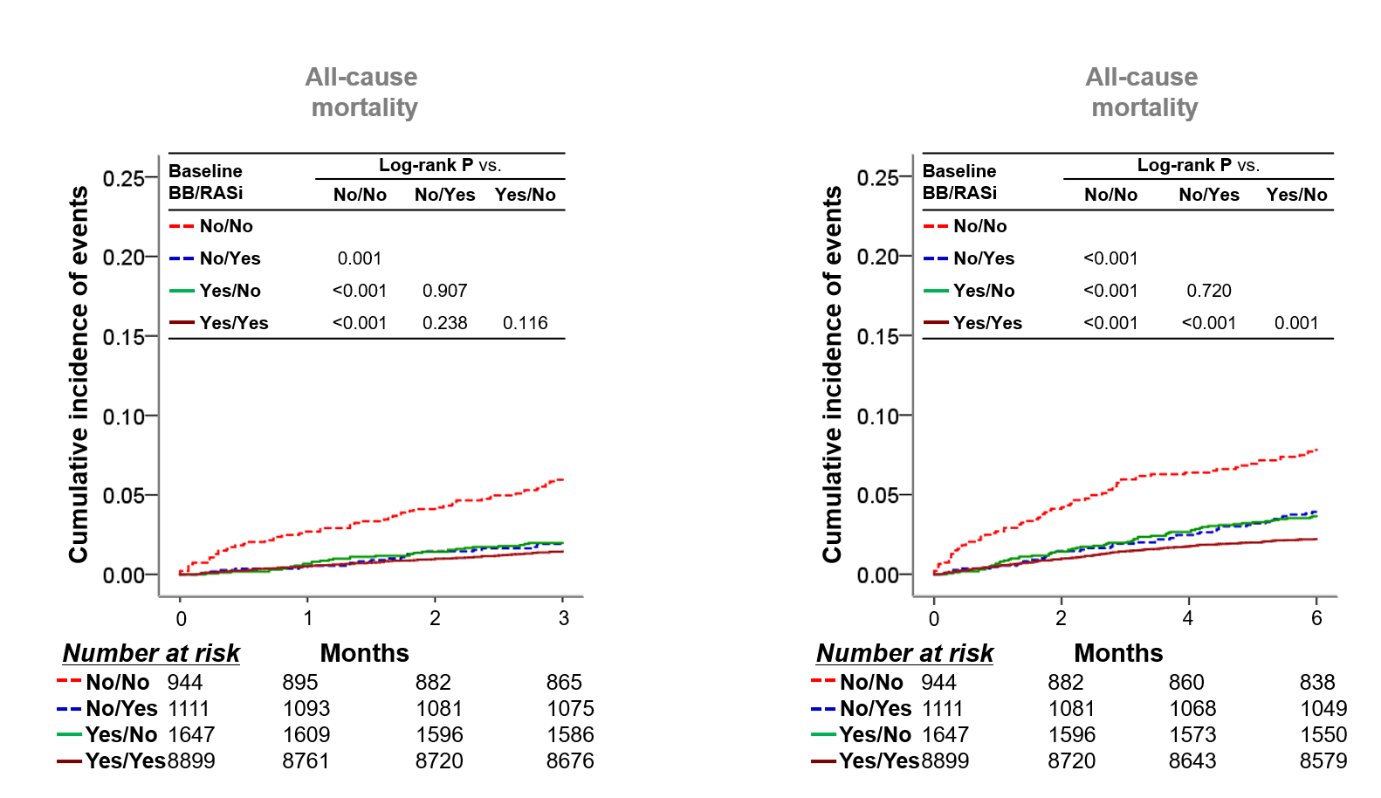


**Supplemental Figure 5. 3-months and 6-months clinical outcomes according to RASi medication and beta-blocker medication at discharge from index hospitalization**

Kaplan-Meier survival curves for 3-months and 6-months post-discharge all-cause mortality and cardiac mortality are presented.

BB: beta-blocker, HR: hazard ratio, RASi: renin-angiotensin-system inhibitor

**Supplemental Table 1. Clinical characteristics based on use of RASi at discharge**

|  | **Without RASi**  **(n=2591)** | **With RASi**  **(n=10010)** | **p-value** |
| --- | --- | --- | --- |
| ***At Index admission*** |  |  |  |
| **Demographic data** |  |  |  |
| Age (years) | 64.4 ± 12.7 | 63.4 ± 12.5 | 0.001 |
| Men (%) | 1933 (74.6) | 7404 (74.0) | 0.509 |
| BMI (kg/m^2^) | 23.7 ± 3.3 | 24.1 ± 3.3 | <0.001 |
| **Past Medical History (%)** |  |  |  |
| Hypertension | 1203 (46.4) | 5182 (51.8) | <0.001 |
| Diabetes mellitus | 741 (28.6) | 2807 (28.0) | 0.574 |
| Dyslipidemia | 309 (11.9) | 1134 (11.3) | 0.395 |
| Myocardial infarction | 205 (7.9) | 779 (7.8) | 0.826 |
| Congestive heart failure | 43 (1.7) | 148 (1.5) | 0.492 |
| Cerebrovascular accident | 180 (7.0) | 63 (6.4) | 0.268 |
| Current Smoking | 972 (38.5) | 4017 (41.1) | 0.019 |
| **Characteristics of lesion and PCI (%)** |  |  |  |
| STEMI | 1147 (44.3) | 4850 (48.5) | <0.001 |
| Complete revascularization | 1435 (70.4) | 6493 (69.2) | 0.2633 |
| **Peak Cardiac enzyme levels** |  |  |  |
| CK-MB (ng/mL) | 46.8 (9.4 - 178.3) | 47.2 (9.3 - 161.2) | 0.296 |
| Troponin I (ng/mL) | 14.6 (2.6 - 46.9) | 16.8 (2.8 - 50.0) | 0.273 |
| **Echocardiography** |  |  |  |
| LVEF (%) | 52.1 ± 11.4 | 52.2 ± 10.9 | 0.594 |
| LVEF <50% (%) | 951 (38.4) | 3631 (37.3) | 0.278 |
| **Physical exam at discharge** |  |  |  |
| SBP (mmHg) | 111.7 ± 15.6 | 113.6 ± 15.0 | <0.001 |
| DBP (mmHg) | 67.1 ± 9.8 | 68.3 ± 9.9 | <0.001 |
| HR (beats per min) | 72.7 ± 11.7 | 70.6 ± 10.4 | <0.001 |
| **Medication at discharge (%)** |  |  |  |
| Beta-blocker | 1647 (63.6) | 8899 (88.9) | <0.001 |
| Statin | 2271 (87.6) | 9494 (94.8) | <0.001 |

BMI: Body mass index, CK-MB: Creatine kinase-myocardial band, DBP: Diastolic blood pressure, HF: Heart failure, HR: Heart rate, LVEF: Left ventricular ejection fraction, RASi: Renin-angiotensin-system inhibitor, SBP: Systolic blood pressure, STEMI: ST-segment elevation myocardial infarction
